# The anterior cingulate cortex mediates cisplatin-induced mechanical allodynia and represents a target for cannabigerol antinociception

**DOI:** 10.1177/17448069261491110

**Published:** 2026-09-15

**Authors:** Matheus Vinicius Ferreira, Nathan Morris, Quinn Wade, Joice Maria da Cunha, Nicholas Graziane

**Affiliations:** 1 Department of Anesthesiology and Perioperative Medicine, 12310 Penn State College of Medicine, Hershey, PA, USA; 2 Department of Pharmacology, Biological Science Building, 28122 Federal University of Paraná, Curitiba, PR, Brazil

**Keywords:** cannabigerol, neuropathic pain, cisplatin, anterior cingulate cortex

## Abstract

Chemotherapy-induced peripheral neuropathy (CIPN) is a prevalent and debilitating consequence of cancer treatment with limited effective therapeutic options. While peripheral nerve injury is a key driver, emerging evidence suggests that maladaptive plasticity within central pain circuits, including the anterior cingulate cortex (ACC), contributes to the maintenance of neuropathic pain. Here, we tested the hypothesis that the ACC is a critical substrate for cisplatin-induced mechanical allodynia and a target for cannabigerol (CBG)-mediated antinociception. Adult male C57BL/6 mice received cisplatin (5 mg/kg, i.p., once weekly for four weeks) to induce CIPN. Mechanical allodynia was assessed using electronic von Frey testing. Systemic administration of CBG (20 mg/kg, i.p.) significantly reversed mechanical allodynia in CIPN mice without affecting baseline thresholds in non-neuropathic animals, indicating a state-dependent effect. Chemogenetic inhibition of ACC neurons using hM4Di DREADDs similarly attenuated mechanical allodynia, identifying the ACC as a functionally relevant component of the CIPN pain state. To determine whether CBG acts within this circuit, bilateral intra-ACC microinjections of CBG (20 nM and 20 µM) were performed, both of which produced transient antinociceptive effects. These findings demonstrate that the ACC contributes to the maintenance of mechanical allodynia in CIPN and establish this region as a site of action for CBG. Together, our results support a model in which the ACC represents a convergent cortical mechanism underlying pathological pain and highlight the potential for centrally targeted, non-euphoriant cannabinoid-based therapies.

## 1. Introduction

Chemotherapy-induced peripheral neuropathy (CIPN) is a prevalent, dose-dependent adverse effect of several antineoplastics, such as taxanes (paclitaxel, docetaxel), vinca alkaloids (vincristine), and platinum-based compounds (cisplatin, oxaliplatin).^1^ A significant portion of patients, estimated at 68%, experience neuropathic pain within the first month of their chemotherapy regimen.^2^ Moreover, even months after treatment cessation, at least 30% of these patients still suffer from persistent symptoms (*e.g.,* numbness, tingling, burning pain in the hands and feet) that significantly impair quality of life.^3^ Therefore, it is critical to define the central nervous system mechanisms that encode and maintain neuropathic pain and to identify targeted therapeutic strategies that can act within these regions.

The anterior cingulate cortex (ACC) encodes the aversive components of pain, largely mediated by pyramidal neurons, located predominantly in layers II/III and V.^4^ These neurons are responsible for projecting to diverse downstream brain regions, including projections to the periaqueductal gray (PAG), which is critical for modulating descending analgesic responses.^5–10^ Furthermore, the ACC maintains reciprocal connections with other cortical areas, such as the somatosensory cortex, allowing the ACC to integrate with the sensory-discriminative properties of the stimulus.^11–13^ Crucially, converging evidence demonstrates that the ACC undergoes maladaptive plasticity in chronic pain states.^14–17^ Peripheral injury induces long-term potentiation and enhanced excitatory synaptic transmission in ACC pyramidal neurons, resulting in persistent cortical hyperactivity that can be maintained independently of ongoing peripheral input.^18,19^ In both inflammatory and neuropathic pain models, ACC pyramidal neurons exhibit increased intrinsic excitability, including elevated firing rates and reduced activation thresholds, consistent with a sensitized cortical state.^16,18^

Functionally, this hyperactivity is not merely correlative but causally linked to pain-like behavior. Selective activation of ACC excitatory neurons is sufficient to enhance mechanical hypersensitivity, whereas inhibition of these neurons reverses pain-like behaviors, including mechanical allodynia in animal models of inflammatory pain.^20^ Together, these findings identify ACC pyramidal neuron hyperexcitability as a key driver of chronic pain states and establish the ACC as a critical supraspinal node in pain signaling.

Notably, current pharmacological approaches for CIPN act centrally, reflecting the importance of supraspinal circuits in pain processing. For example, serotonin-norepinephrine reuptake inhibitors (SNRIs) have shown promise for reducing neuropathy pain.^21^ However, side effects often compromise treatment adherence,^1,22^ creating an urgent need for novel therapeutic compounds with fewer side effects and a rapid onset of action.

In this context, phytocannabinoids have emerged as promising candidates.^23,24^ While most research has historically focused on Δ9-tetrahydrocannabinol (THC) and cannabidiol (CBD), *Cannabis sativa* produces over 100 other bioactive compounds, including Cannabigerol (CBG). CBG is the decarboxylated form of cannabigerolic acid, the primary scaffold for the biosynthesis of both acidic forms of THC and CBD.^25^ Importantly, unlike THC, CBG is considered non-euphoriant and lacks the canonical psychoactive effects associated with CB1 receptor activation,^26^ supporting its potential as a therapeutically relevant compound with a more favorable side effect profile.

Beyond its biosynthetic role, CBG exhibits a complex and somewhat unconventional pharmacological profile, including high-affinity α2-adrenoceptor agonist and a 5-HT1A receptor antagonism in addition to acting as a CB1 and CB2 receptor partial agonist.^27^ Behavioral evidence suggests that CBG-mediated antinociception is contingent upon the functional integrity of CB1, CB2, and α2-adrenergic receptor signaling pathways.^28–30^ However, the specific neural substrates required for CBG’s antinociceptive effects remain largely unknown, particularly within the supraspinal circuits that drive the affective-sensory components of CIPN.

Therefore, in this study, we tested the hypothesis that the ACC is a necessary hub for the expression of cisplatin-induced mechanical hypersensitivity and that CBG modulates this circuit. By combining chemogenetics, and site-specific microinjections, we aimed to dissect if CBG’s antinociceptive effects are mediated in ACC.

## 2. Methods

### 2.1. Animals

All experiments were done in accordance with procedures approved by the Pennsylvania State University College of Medicine Institutional Animal Care and Use Committee. Mice used in this study included male C57BL/6 wild-type mice (The Jackson Laboratory, Bar Harbor, ME) (N = 109). 12 mice were excluded from final analyses due to failure to develop confirmed cisplatin-induced mechanical allodynia or inaccurate stereotaxic targeting determined by post-mortem verification of viral expression or cannula placement, resulting in a final analyzed cohort of 97 mice. All mice were group-housed on a 12-h light/dark cycle with ad libitum food and water. Mice used for behavioral assessments were aged approximately 8-12 weeks. Random placement of home cages within the housing and behavioral rooms was employed for behavioral experiments to control any environmental factors (*e.g.,* room lighting, vibrations).^31^

### 2.2. Drugs and doses

Cisplatin solution was purchased from Acros Organics (Fairlawn, NJ). Indomethacin was purchased from Alfa Aesar (Haverhill, MA). Indomethacin was included as a reference anti-inflammatory comparator, which may attenuate, but does not necessarily fully reverse mechanical hypersensitivity in neuropathic pain models.^32,33^ Cannabigerol (CBG) was purchased from Cayman Chemical (Ann Arbor, MI). Clozapine N-Oxide (CNO) was provided by the National Institutes of Health (NIH; Bethesda, MD). CBG, indomethacin, CNO and VEH were diluted in a mixture of DMSO, Tween 80 and saline (1:1:18). Cisplatin was diluted in a sterile 0.9% saline solution.

The systemic dose of CBG (20 mg/kg, i.p.) was selected based on previous findings of our lab showing peak plasma concentrations and maximal efficacy in increasing mechanical thresholds.^29^ The indomethacin dose (10 mg/kg, i.p.) was likewise determined from prior studies demonstrating significant antinociceptive effects.^29^ For chemogenetic experiments, CNO (3 mg/kg, i.p.) was administered at a dose previously validated to ensure DREADD activation without inducing sedation or affecting baseline mechanical thresholds.^34^ Intra-ACC CBG concentrations (20 nM and 20 μM) were selected based on prior pharmacokinetic studies, which show that intraperitoneal administration of CBG (120 mg/kg) produces brain concentrations on the order of micrograms per gram, with peak levels occurring within the first 1–2 hours and declining toward baseline within 24 hours.^35^ These doses/concentrations were chosen to approximate physiologically relevant low- and high-exposure conditions.

### 2.3. CIPN model

Cisplatin-induced peripheral neuropathy was established using previously described protocols.^36,37^ C57BL/6 wild-type mice received intraperitoneal injections of cisplatin (5 mg/kg) or vehicle (0.9% saline) once per week for 4 consecutive weeks. To reduce the risk of nephrotoxicity and protect renal function, a 4% sodium bicarbonate solution (400 μL, subcutaneously) was administered after each cisplatin dose, as outlined previously.^36^ Mechanical allodynia was evaluated before and after treatment to confirm the development of neuropathic pain.

### 2.4. Electronic von frey test

Mechanical hypersensitivity was evaluated using an electronic von Frey aesthesiometer (IITC Life Sciences Inc., Woodland Hills, CA, USA). Mice were placed in one of six raised individual runs (11 cm × 4 cm × 11.5 cm) equipped with Plexiglas sides and steel rod floors, sized and spaced to accommodate mouse paws. Animals were allowed to acclimate to the runs for 20 minutes prior to testing. The aesthesiometer, fitted with a rigid tip, was applied to the plantar surface of the right hind paw with gradually increasing force to elicit a paw withdrawal response. Each animal underwent three measurements on the right hind paw, with at least three minutes between trials, and the average withdrawal threshold was calculated.

### 2.5. Stereotaxic surgery

Anesthesia was induced and maintained with 1.5% isoflurane. The animal was placed in a stereotaxic frame (Stoelting) and craniotomies were performed via microdrill. Injections were carried out via a 33-gauge beveled-tip needle (WPI) connected to a 5 μl syringe (Hamilton) on a micro pump (Harvard Apparatus) at an infusion rate of 100 nL/min for 3 min. Following bilateral injection, the needle was left *in situ* for 5-10 minutes to allow for virus diffusion and then slowly retracted to limit backflow. For chemogenetic experiments, AAV5-hSyn-hM4D (Gi)-mCherry (Addgene #50475, titer: 8.6 x 10^12^ GC/mL) was injected bilaterally into the ACC (coordinates relative to Bregma, anterior-posterior (AP) +1.10 mm; medial lateral (ML) ±0.25mm; dorsal ventral (DV) -2.00 mm). AAV5-hSyn-hM4D (Gi)-mCherry was a gift from Bryan Roth. Sham-operated animals underwent the same stereotaxic procedure, including craniotomy and bilateral needle placement within the ACC, but did not receive the viral vector.

### 2.6. ACC chemogenetic inhibition

To assess the contribution of ACC neuronal activity to mechanical hypersensitivity in the CIPN model, we used the Gi-coupled hM4Di DREADD. After five to six weeks of viral expression, hM4Di-expressing mice received CNO (3 mg/kg, i.p.) or vehicle, whereas sham-operated mice received CNO (3 mg/kg, i.p.). Mechanical withdrawal thresholds were assessed 30 min, 60 min, and 72 h after injection. To verify injection sites, acute brain slices were placed on the electrophysiology rig and imaged using an Olympus BX51WI microscope equipped with epifluorescence optics, a Retiga 3.0 digital camera (QImaging) and Ocular software. Images were acquired with a 100 ms exposure and 100% fluorescence intensity. No immunofluorescent signal amplification was performed; native mCherry fluorescence was imaged directly.

### 2.7. Cannula implantation

One week prior to the testing intra-ACC injection of the treatment compounds, mice were implanted with bilateral 26-gauge guide cannulae (PlasticsOne) targeting the ACC. The following stereotaxic coordinates, relative to the bregma, were used: AP, +1.10 mm; ML, ±0.25 mm; and DV, –1.00 mm. The cannulae were secured to the skull using dental cement. For the microinjections, a 33-gauge internal needle extending 1 mm beyond the tip of the guide cannula (reaching a total depth of 2.00 mm) was used to deliver a volume of 200 nL per side of testing compounds.

For intra-ACC experiments, mice were tested across two sessions separated by a 7-day washout period. Control mice received vehicle in both sessions. A separate cohort received 20 nM CBG in the first session followed by 20 µM CBG in the second session, corresponding to approximately 1.26 pg or 1.26 ng delivered per hemisphere, respectively. This design was selected to preserve an uncontaminated vehicle control condition, since prior exposure to CBG, even at the lower concentration, could potentially influence subsequent behavioral responses. The fixed low-to-high concentration sequence also minimized the possibility that exposure to the higher concentration would carry over into later low-concentration testing.

Upon completion of the behavioral assessments, cannula placements were verified by injecting 5 mM Evans blue (MP Biomedicals) through the guide cannula. The infusion rate for the dye was consistent with the rate used for drug delivery during the experimentation. Brain slices were subsequently prepared on a vibratome to identify the precise anatomical location and the diffusion of the dye through the ACC tissue. Only data from mice with confirmed bilateral placement within the targeted region were included in the statistical analysis. One animal was excluded because of inaccurate cannula placement.

### 2.8. Statistical analyses

The Shapiro-Wilk normality test was used on all data. Since the data followed a Gaussian distribution, they are presented as the mean ± standard error of the mean (SEM). To identify differences among experimental groups, when suitable, we performed one-way or two-way ANOVA, followed by Bonferroni’s post hoc test. For repeated-measures ANOVA, violations of the sphericity assumption were assessed in GraphPad Prism, and Greenhouse–Geisser correction was applied when violations were detected, yielding a more conservative test by adjusting the degrees of freedom to control inflation of Type I error. For ANOVA analyses, *F*-values are reported for main effects and interaction terms, as indicated in the Results. Effect sizes are reported as eta squared (η^2^), defined as the proportion of total variance explained by each factor. Effect sizes are reported to complement exact *p*-values by providing a scale-independent measure of the magnitude of observed effects, consistent with contemporary statistical guidance emphasizing that *p*-values alone do not convey effect size, practical relevance, or biological importance.^38^ For interpretive context, η^2^ values of approximately 0.01, 0.06, and 0.14 are often considered small, medium, and large effects, respectively,^39,40^ although these benchmarks are provided as general guidelines and interpretation was informed by experimental context. Additionally, 95% confidence intervals (CIs) are provided for group means, mean differences, or model estimates, where applicable, to convey estimation uncertainty and complement *p*-values and effect size measures.

A significance level of *p* < 0.05 was established. All statistical analyses were performed with GraphPad Prism version 11.0.1 (GraphPad Software, San Diego, CA, USA).

## 3. Results

### 3.1. CBG attenuates mechanical hypersensitivity in CIPN male mice

**Figure 1.**
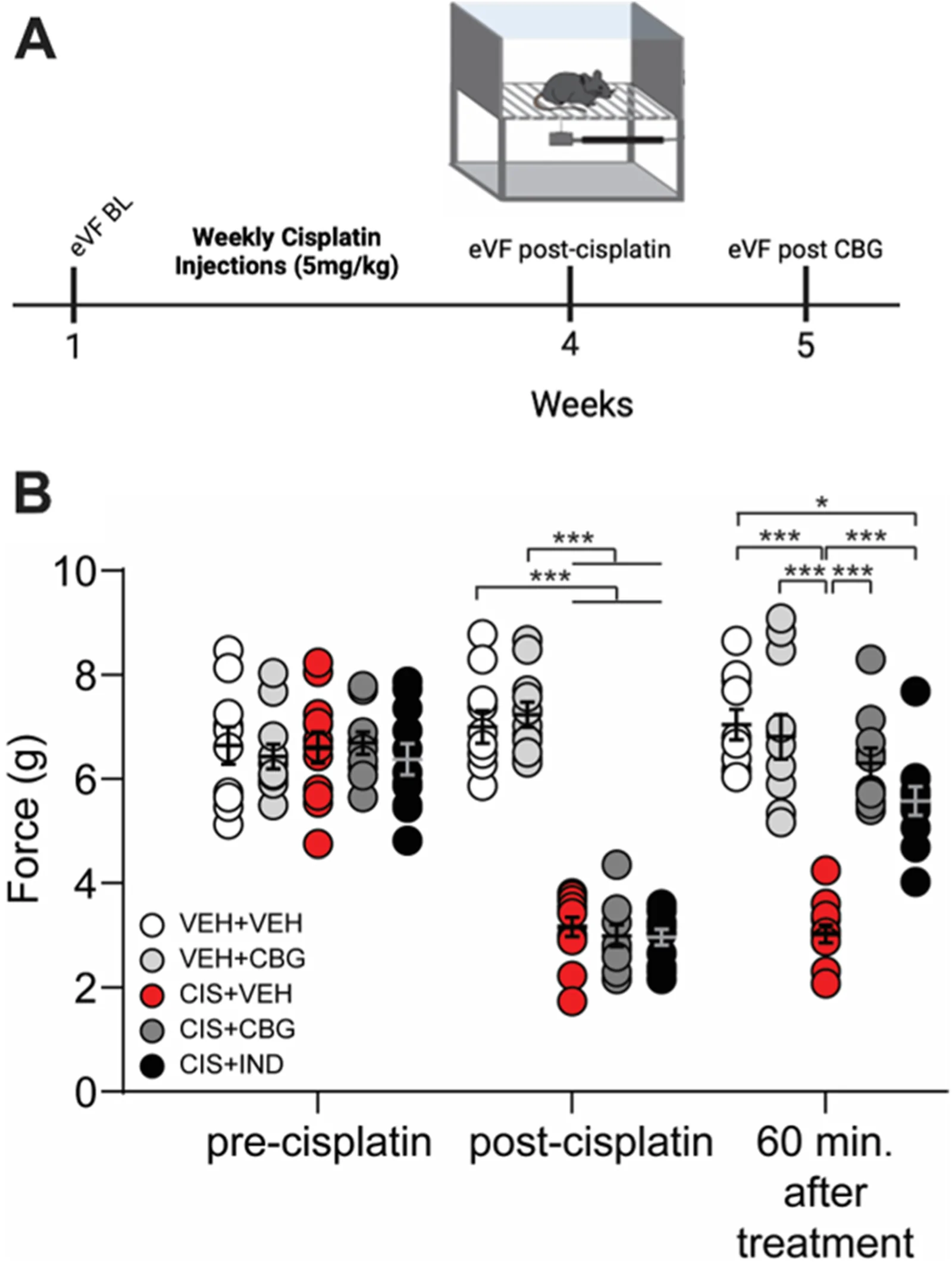
CBG attenuates mechanical hypersensitivity in CIPN male mice. (A) Schematic of the experimental design. Male mice received weekly intraperitoneal injections of cisplatin (5 mg/kg) for 4 weeks. One week after confirming the development of mechanical hypersensitivity (reduced mechanical threshold), animals were treated acutely with vehicle, CBG (20 mg/kg, i.p.), or the comparator indomethacin (IND, 10 mg/kg, i.p.), followed by behavioral testing 60 minutes post-treatment. (B) Mechanical threshold was assessed using the electronic von Frey test and expressed as force (g). Data are presented as mean ± SEM. *p < 0.05, ***p < 0.0001. n = 10-12 animals per group.

To investigate whether CBG has a behavioral effect in a mouse model of CIPN, male mice received intraperitoneal injections of cisplatin (5 mg/kg) once a week for 4 consecutive weeks (Figure 1(a)). Mechanical thresholds were assessed using electronic von Frey testing at baseline, after the final cisplatin administration, and following treatment with either CBG, indomethacin (a comparator), or vehicle. Prior to treatment, CIPN mice exhibited a significant reduction in mechanical threshold compared to naïve groups (VEH+VEH and VEH+CBG), as shown by a two-way repeated measures ANOVA main effect (*F*_(4, 49)_ = 54.79, *p* < 0.0001, η^2^ = 0.32), time (*F*_(1.848, 90.56)_ = 60.03; *p* < 0.0001, η^2^ = 0.17), and group × time interaction (*F*_(7.392, 90.56)_ = 24.63; *p* < 0.0001, η^2^ = 0.28) (Figure 1(b)).

To determine the effect of treatment on mechanical allodynia, Bonferroni post hoc tests were performed. CBG treatment significantly increased mechanical thresholds in CIPN mice when compared to the CIS+VEH group (mean difference = −3.28, 95% CI [−4.38, -2.17], *p <* 0.0001), effectively reversing mechanical allodynia (Figure 1(b)). Moreover, mechanical thresholds in CBG-treated CIPN mice were not significantly different from naïve controls (mean difference = 0.74, 95% CI [−0.58, 2.06], *p* = 0.8989) (Figure 1(b)), supporting the recovery of baseline sensitivity. Indomethacin treatment also significantly increased mechanical thresholds compared to CIS+VEH (mean difference = −2.55, 95% CI [−3.60, -1.50], *p <* 0.0001). However, thresholds remained significantly lower than in naïve groups, suggesting only partial reversal (Figure 1(b)). Importantly, CBG did not affect mechanical thresholds in naïve mice, as no difference was observed between VEH+CBG and VEH+VEH groups (mean difference = 0.2353, 95% CI [−1.44, 1.91], *p >* 0.9999). These findings replicate and extend previous observations showing that CBG reverses cisplatin-induced mechanical allodynia in male mice.^29^

### 3.2. ACC inhibition attenuates mechanical hypersensitivity in CIPN male mice

**Figure 2.**
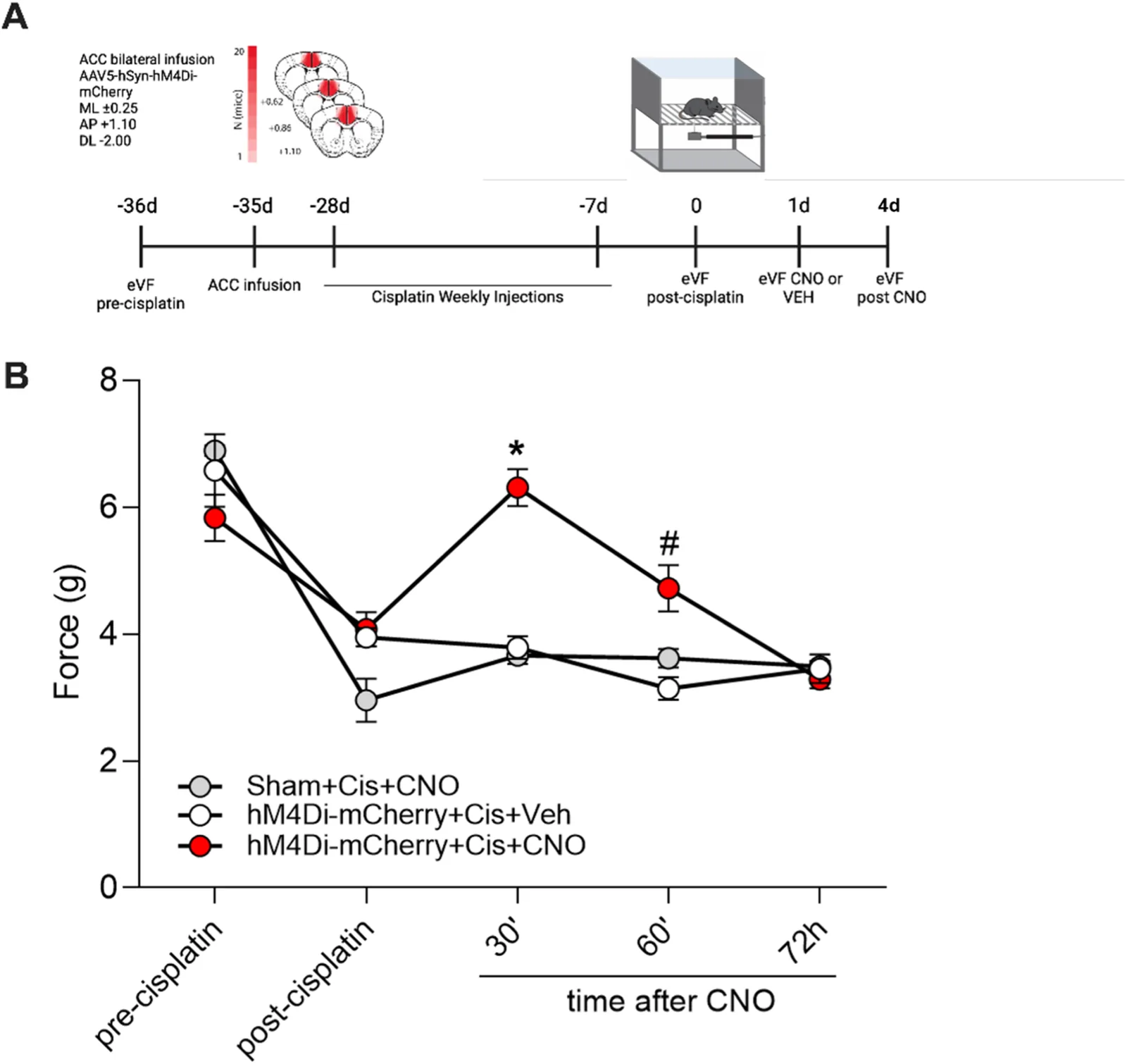
ACC inhibition attenuates mechanical hypersensitivity in CIPN. (A) Experimental timeline. Mechanical withdrawal thresholds were measured using the electronic von Frey (eVF) assay before cisplatin treatment (baseline). Mice received bilateral ACC infusions of AAV5-hSyn-hM4Di-mCherry or sham surgery. Representative distribution of mCherry expression within the ACC is shown, with color intensity indicating the number of animals exhibiting expression at each location. Following a one-week recovery period, mice received weekly intraperitoneal injections of cisplatin (5 mg/kg) for four consecutive weeks. Mechanical withdrawal thresholds were reassessed to confirm the development of neuropathy. One day later, mice received an acute intraperitoneal injection of CNO (3 mg/kg) or vehicle, and eVF testing was performed 30 min, 60 min, and 72 h after treatment. (B) Mechanical threshold (force in grams) at baseline, post-cisplatin, and after acute treatment with CNO (3 mg/kg; i.p.) or VEH. Three experimental groups were evaluated: hM4Di-mCherry + Cis + Veh, hM4Di-mCherry + Cis + CNO, and Sham + Cis + CNO. Data were analyzed by two-way repeated measures ANOVA followed by Bonferroni post hoc tests. *p < 0.05 versus hM4Di + CIS + VEH and Sham + Cis + CNO at the corresponding time point. #*p* < 0.05 versus hM4Di + CIS + VEH at the corresponding time point. n = 8-9 per group.

To further investigate the role of the ACC in CIPN male mice, we employed DREADD-mediated neuronal inhibition, which was activated via systemic injection of CNO (3 mg/kg; i.p.) (Figure 2(a)). As shown in Figure 2(b), cisplatin treatment significantly reduced the mechanical threshold in all experimental groups. Two-way ANOVA revealed significant main effects of group (*F*_(2, 22)_ = 5.950; *p* = 0.0086, η^2^ = 0.04), and time (*F*_(2.834, 62.35)_ = 68.79; *p* < 0.0001, η^2^ = 0.54), as well as a significant group x time interaction (*F*_(5.668, 62.35)_ = 10.12; *p* < 0.0001, η^2^ = 0.16). Bonferroni post hoc comparisons showed that cisplatin significantly reduced mechanical withdrawal thresholds relative to the corresponding pre-cisplatin baseline in all groups (*p* < 0.0001 for all groups).

Thirty minutes after CNO or vehicle administration, mechanical thresholds were significantly higher in hM4Di-expressing mice treated with CNO compared to hM4Di-expressing mice treated with vehicle (mean difference = -2.528, 95% CI [−3.48, -1.58], *p* < 0.0001) and sham mice treated with CNO (mean difference = -2.518, 95% CI [-3.46, -1.57], *p* < 0.0001).

Sixty minutes after CNO or vehicle administration, mechanical withdrawal thresholds remained significantly higher in the hM4Di + Cis + CNO group compared to the hM4Di + Cis + Veh group (mean difference = -1.579, 95% CI [−2.76, -0.40], *p* = 0.0096). However, the difference between hM4Di + Cis + CNO and sham + Cis + CNO did not reach statistical significance (mean difference = -1.071, 95% CI [−2.23, 0.09], *p* = 0.0717).

Seventy-two hours after CNO or vehicle administration, no significant differences were observed between groups (hM4Di + Cis+ Veh vs. hM4Di + Cis + CNO: mean difference = 0.1650, 95% CI [−0.59, 0.92], *p* > 0.9999; sham + Cis + CNO vs. hM4Di + Cis + CNO: mean difference = 0.3032, 95% CI [−0.21, 0.82], *p* = 0.3935).

### 3.3. Local ACC injection of CBG attenuates mechanical hypersensitivity in CIPN male mice

**Figure 3.**
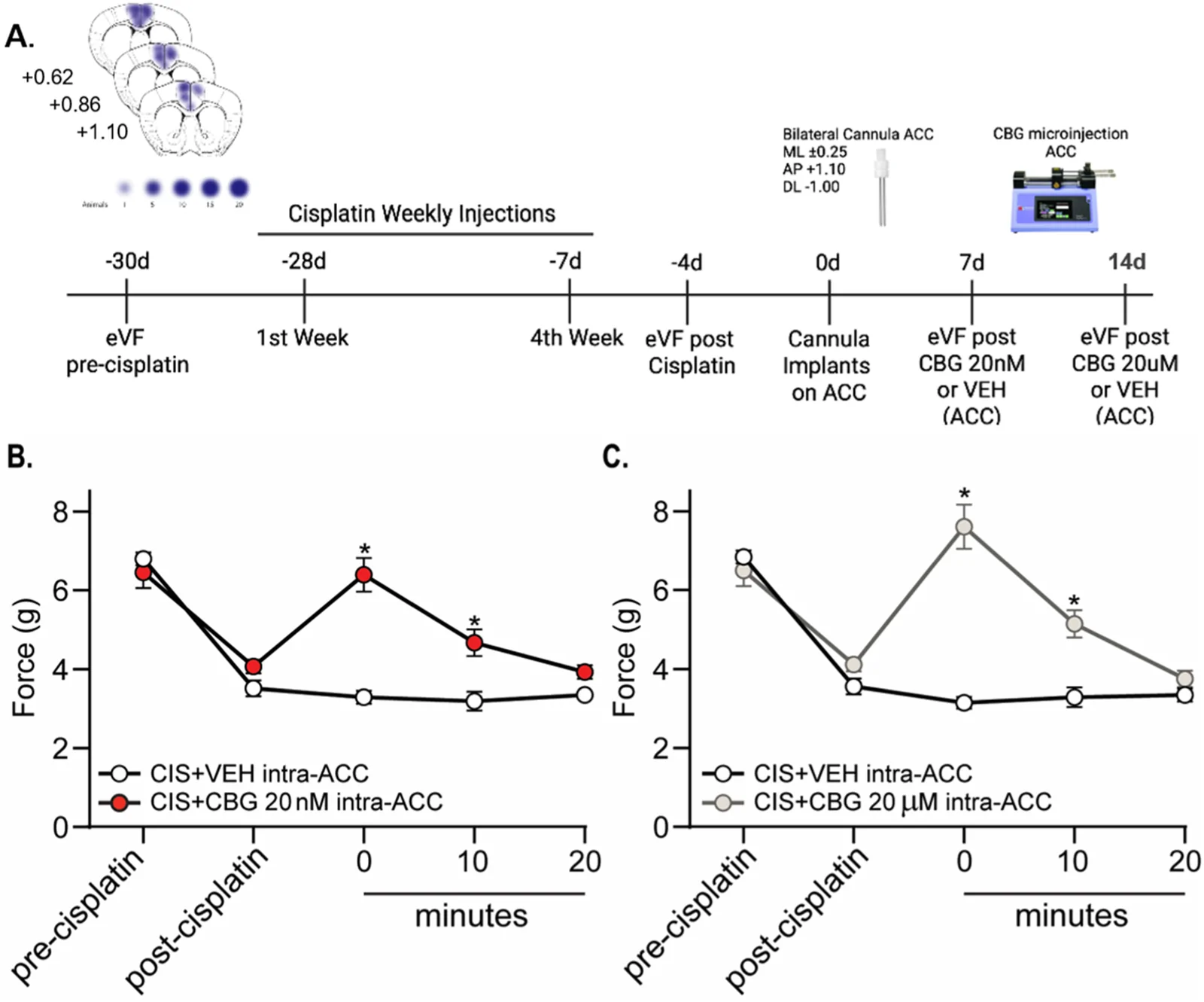
Local ACC injection of CBG attenuates mechanical hypersensitivity in CIPN. (A) Experimental timeline. Baseline mechanical sensitivity was assessed using electronic von Frey (eVF), followed the next day by the initiation of weekly intraperitoneal cisplatin injections (5 mg/kg) for 4 consecutive weeks. Mechanical thresholds were reassessed after the final injection to confirm the establishment of neuropathy. The following day (referred to as day 0), mice underwent stereotaxic cannula implantation targeting the ACC (Top left: Summary of ACC cannula placements. The figure represents Evans Blue staining of individual animals in coronal brain sections (from bregma: +0.62 to +1.10). Intensity of the stain is based on the number of animals with an N of 1 corresponding to low intensity and N of 20 the maximum intensity). After 1 week of recovery, animals received intra-ACC microinjections of either vehicle or CBG at 20 nM or 20 µM with a fixed low-to-high concentration sequence. A 7-day washout period was included between sessions. (B) Intra-ACC microinjections of either vehicle or CBG at 20 nM; mechanical threshold (force in grams) measured at baseline, post-cisplatin, and immediately (0 minutes), 10, and 20 minutes following intra-ACC microinjection. (C) Intra-ACC microinjections of either vehicle or CBG at 20 µM; mechanical threshold (force in grams) measured at baseline, post-cisplatin, and immediately (0 minutes), 10, and 20 minutes following intra-ACC microinjection. Data was analyzed by two-way repeated measures ANOVA followed by Bonferroni post hoc test. *p < 0.05 vs. CIS+VEH at the same timepoint. n = 9 per group.

To evaluate whether local administration of CBG into the ACC modulates cisplatin-induced mechanical allodynia, we bilaterally microinjected two concentrations of CBG (20 nM and 20 µM) into the ACC of CIPN male mice. Mice were tested across two intra-ACC injection sessions separated by a 7-day washout period. Vehicle-control mice received vehicle in both sessions, whereas CBG-treated mice received 20 nM CBG in the first session and 20 µM CBG in the second session. As experimental controls, the same animals were tested before cisplatin (pre-cisplatin), after cisplatin (post-cisplatin), and following intra-ACC vehicle injection (CIS+VEH) (Figure 3(a)).

As shown in Figure 3(b), CBG at 20 nM produced an increase in mechanical threshold (two-way ANOVA main effect of treatment: *F*_(1, 16)_ = 42.77; p<0.0001, η^2^ = 0.12; main effect of time: *F*_(2.685,42.96)_ = 45.16; *p* < 0.0001, η^2^ = 0.51) and an interaction between group × time (*F*_(2.685,42.96)_ = 12.44; *p* < 0.0001, η^2^ = 0.14). Bonferroni post hoc analysis revealed a significant effect immediately following injection (0 minute) (mean difference = -3.102, 95% CI [−4.56, -1.64], *p* = 0.0002), and 10 minutes after injection (mean difference = -1.476, 95% CI [−2.72, -0.23], *p* = 0.0165) compared to the CIS+VEH group. No significant differences were detected at 20-minute post-injection (mean difference = -0.5756, 95% CI [−1.17, 0.02], *p* = 0.0600).

Similarly, Figure 3(c) shows that intra-ACC injection of 20 µM CBG induced an increase in mechanical threshold as shown by two-way ANOVA main effects (*F*_(1, 16)_ = 32.74; p<0.0001, η^2^ = 0.15), time (*F*_(2.623,41.98)_ = 47.44; *p* < 0.0001, η^2^ = 0.41) and interaction (*F*_(2.623,41.98)_ = 25.68; *p* < 0.0001, η^2^ = 0.22). Post hoc comparisons indicated significant analgesia immediately following injection (0 minutes) (mean difference = -4.468, 95% CI [−6.36, -2.57], *p* = 0.0001) and 10 minutes (mean difference = -1.864, 95% CI [−3.15, -0.58], *p* = 0.0034) post-injection compared to CIS+VEH, with the peak effect at time 0. By 20 minutes, the effect was no longer significant (mean difference = -0.4033, 95% CI [−1.19, 0.39], *p* = 0.7686).

Together, these findings indicate that intra-ACC CBG rapidly attenuates cisplatin-induced mechanical allodynia in male mice. However, this effect was transient, peaking immediately after microinjection and dissipating by 20 minutes, suggesting that CBG produces short-lived modulation of ACC-dependent pain processing rather than sustained reversal of CIPN-related sensitization.

## 4. Discussion

Our study provides evidence that the ACC is a neural substrate in the maintenance of mechanical allodynia in a mouse model of CIPN. By employing a multidisciplinary approach, we demonstrated that chemogenetic inhibition of the ACC effectively reverses mechanical allodynia, suggesting this region is a functionally relevant component of the CIPN pain state. Furthermore, we identified CBG as an antinociceptive agent that acts in a state-dependent manner, attenuating pain in CIPN mice while sparing normal mechanical sensory thresholds in non-neuropathic mice. Crucially, the local administration of CBG directly into the ACC mirrored its systemic effects, identifying this cortical area as a key neural substrate for its therapeutic properties.

Our findings are consistent with prior studies, which demonstrate that chemogenetic or optogenetic inhibition of ACC excitatory neurons alleviates mechanical hypersensitivity in an animal model of inflammatory pain.^19^ However, because the viral approach used in the present study was not designed to selectively target excitatory neurons, our findings should be interpreted as evidence that broader ACC neuronal inhibition attenuates mechanical allodynia. The extension of this finding to CIPN supports the ACC as a convergent cortical substrate underlying mechanical hypersensitivity rather than a model-specific phenomenon. Additionally, our results replicate findings that a single systemic administration of CBG effectively reverses mechanical allodynia in a murine model of CIPN.^29,30,41^ Notably, this effect was state-dependent, as CBG did not alter nociceptive thresholds in non-neuropathic mice, suggesting that its action is contingent upon the pathological reorganization of pain-processing circuits.

Guided by the need to identify targeted interventions that act within central pain-processing circuits, our results suggest that CBG can exert antinociceptive effects directly within the ACC. Notably, intra-ACC administration of CBG at 20 nM and 20 μM produced qualitatively similar behavioral effects despite a 1000-fold difference in nominal concentration. One possible explanation is that the relevant ACC mechanism is engaged at low nanomolar concentrations, such that 20 nM may be sufficient to approach a functional ceiling for this behavioral endpoint. In this scenario, increasing the local concentration to 20 μM would not be expected to further enhance antinociception if downstream circuit output is already maximally modulated. Alternatively, the effective concentration at cellular targets may be influenced by local diffusion, tissue binding, or clearance, such that the injected concentrations do not scale linearly with receptor-level exposure. These possibilities remain speculative and will require direct pharmacological and circuit-level analyses to resolve.

Additionally, the efficacy of CBG in the ACC at a 20 nM and 20 μM concentration is significantly lower than the 100 μM concentration previously required for antinociception in the S1HL^28^ and highlights a pronounced regional specialization in CBG sensitivity. The rapid onset of action following intra-ACC infusion, with peak effects observed shortly after injection, is consistent with the signaling kinetics of G_i/o_-coupled GPCRs,^42^ although not directly tested here. It is well-established that CBG activates several such targets, including CB1R and CB2R,^43^ as well as α2-adrenergic receptors.^27–30,44^ The rapid activation and subsequent desensitization of these GPCR pathways are known to acutely modulate neuronal activity,^45,46^ consistent with the transient behavioral effects observed following intra-ACC infusion. This short duration of action also reflects the physicochemical properties of CBG as its high lipophilicity^47^ favors rapid membrane permeation and microvascular uptake, predicting fast local clearance from brain tissue following microinfusion.^35,48–50^ By contrast, systemic administration requires blood-brain barrier transit and broader pharmacokinetic distribution, accounting for its comparatively slower onset. Together, the sensitivity of the ACC to nanomolar concentrations of CBG, along with its diffusion–clearance profile, suggests that this region may represent an important cortical substrate within CBG’s therapeutic window. However, dedicated pharmacokinetic studies will be necessary to directly characterize CBG’s distribution, local concentration, and clearance dynamics in the ACC.

Overall, the present study is not without limitations. Our experiments were conducted exclusively in male mice, whereas it is well-established that female subjects exhibit distinct patterns of nociceptive processing and neuroimmune responses.^51^ Therefore, future research must incorporate sex as a biological variable to determine whether the functional necessity of the ACC and the therapeutic efficacy of CBG are preserved in female subjects within the context of cisplatin-CIPN. In addition, although two intra-ACC CBG concentrations produced similar antinociceptive effects, the present study did not include a comprehensive concentration-response analysis or pharmacological dissection of the underlying receptor mechanisms. Consequently, the explanations proposed here remain speculative and should be addressed in future studies.

In conclusion, this study identifies the ACC as a hub in the maintenance of mechanical allodynia in a model of CIPN. By demonstrating that chemogenetic inhibition of the ACC reverses hypersensitivity and that both systemic and local administration of CBG produce antinociceptive effects, our findings link circuit-level dysfunction to a pharmacologically targetable mechanism within this region. These results support a model in which increased or dysregulated ACC neuronal activity may represent a convergent feature of pathological pain states. Importantly, these findings demonstrate that CBG can act within the ACC to modulate mechanical allodynia. Future studies identifying the receptor targets and circuit mechanisms through which CBG acts within the ACC will be essential for refining therapeutic strategies for chronic pain.

## Data Availability

The authors declare that all data supporting the findings of this study are available within the manuscript and its Supplementary Data. Additional data and materials are available from the corresponding authors upon reasonable request.*
